# Comparing the conventional 15 cm and the C-length approaches for central venous catheter placement

**DOI:** 10.15171/jcvtr.2018.38

**Published:** 2018-12-11

**Authors:** Hashem Jarineshin, Maryam Sharifi, Saeid Kashani

**Affiliations:** Anesthesiology, Critical Care and Pain Management Research Center, Hormozgan University of Medical Sciences. Bandar Abbas, Iran

**Keywords:** Catheterization, Central Venous, Vena Cava Superior, Radiography

## Abstract

***Introduction:*** The present guidelines recommend placing the catheter tip in the superior vena cava (SVC) above the pericardial cephalic reflection. The aim of this study was to compare the accuracy of two different approaches in locating the tip of the Central venous catheter (CVC) at the suggested vascular zone.

***Methods:*** This was an interventional study on two hundred patients undergoing Coronary artery bypass surgery (CABG) operation who required a central venous cannulation. They were randomly assigned into two groups. In the first group catheter placement was applied through using the conventional 15 cm method. In the second group a C-length method was applied for measuring the depth of catheter tip placement from the preoperative chest radiographs. For statistical analysis Chi-square test and T-test were used.

***Results:*** In the first group (15 cm) 100% of the patients had their catheters placed below the C-line (Carina line) and the average distance between the catheter tip and the C-line was +4.22±2.10 cm. In the second (C-Length) group 52% of the catheters were below C-line with an average distance of +0.77±0.5 cm. There was a meaningful difference between the two groups in respect to the catheter location depth and zone of placement (*P*<0.001).

***Conclusion:*** The C-Length approach in comparison to the conventional 15 cm approach resulted in a considerable higher number of catheters above the recommended C-line, thus it can provide a more reliable and safe mode for CVC placement in the SVC.

## Introduction

Central vein cannulation and direct measurement of central venous pressure (CVP) are frequently performed in hemodynamically unstable patients and those undergoing major operations. A central venous catheter (CVC) may be inserted to provide secure vascular access for many reasons such as administration of vasoactive drugs or fluids, CVP monitoring, transvenous cardiac pacing, temporary hemodialysis, pulmonary artery catheterization, or for repeated blood sampling.^1^

CVCs are commonly inserted into the right and left internal and external jugular veins, right and left subclavian veins and femoral veins. The incorrect positioning may give false CVP readings leading to incorrect volume replacement and cause other serious complications such as vascular laceration, hemothorax, hydrothorax, pneumothorax, arrhythmias, placement in the coronary sinus, tricuspid valve damage, thromboemboli, infection, nerve damage, pressure on the airway by hematoma, tracheal injury and cardiac tamponade and even death.^1-4^ Unsuitable CVC tip placement may lead to inaccurate CVP measurements and consequently inappropriate fluid therapy may cause inadequate hemodynamic control and its complications.^5^

A simple anatomic topographical method for accurate CVC placement should decrease serious complications of CVC insertion.^5^ Current guidelines recommend that the tip of a CVC be located in the superior vena cava (SVC).^6^ Food and Drug Administration (FDA) guidelines regarding proper catheter tip placement strongly require that no catheter tips should be located into the heart in other wards it must be located outside the right atrium to avoid damaging the heart^7^ or to minimize the risk of cardiac tamponade, it has been suggested that CVC tips should not be within the boundaries of the pericardial sac in other words to be located above the cephalic limit of the pericardial reflection, not merely at the SVC/right atrium junction.^7-9^

So far, different techniques have been used for evaluating the appropriate location of the CVC tip such as CXR, ECG or endo-cavitary electrocardiography, sonography and fluoroscopy^2,3,10,11^ among which the fluoroscopy guided CVC placement is the most accurate method but, in a busy setting of the operating or emergency room conditions they are time consuming and may not be easily available.^12^

A post-procedural posterior-anterior chest x-rays (PACXR) is a reliable, easily available and applicable method to confirm placement of the CVC tip in the SVC above the pericardial reflection. The upper limit of the pericardial reflection cannot be seen on a plain chest radiograph (CXR), however it is generally accepted that it is below the carina.^4^ Therefore carina, which is located in the mid-zone of the SVC, can be considered as a useful and accurate radiological landmark to estimate the position of CVC tip relative to the pericardial reflection.^10^

Different methods of estimating the depth of CVC insertion have been published.^5,13,14^

**Figure 1:**
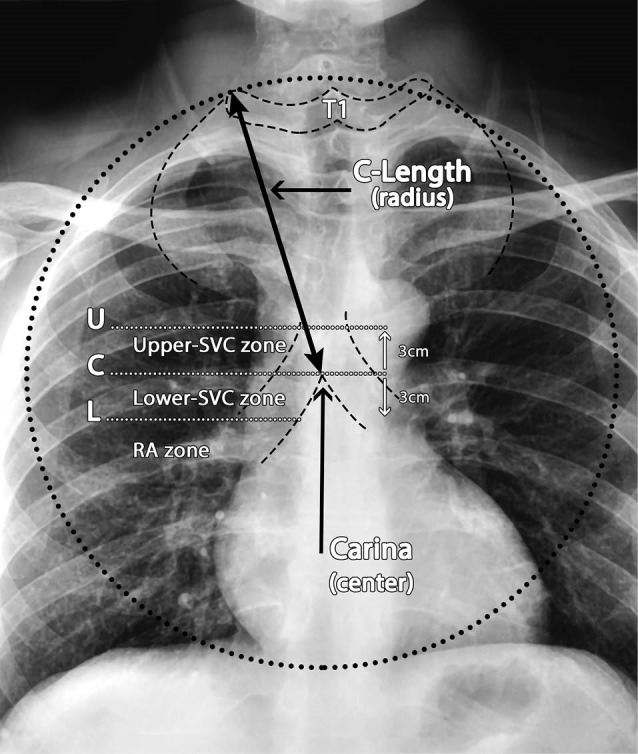
The PA CXR was assessed before operation in the C-Length and after operation for both groups. A circle which was drawn from the focal point of carina as its boundary crossed the tip of the edge of the transverse processes of the first thoracic vertebra. A dashed line C (central carinal line), dashed line U (upper line), and dashed line L (lower line as the cavoatrial junction). Line U and line L were respectively defined as 3 cm above and below the C line. Line U-C (upper-SVC or safe zone), line C-L (lower-SVC or intermediate zone) and below line L (RA or unsafe zone).

A novel method introduced by Lee and Lee^10^ using two radiographic landmarks on the PA CXR for identifying the length at which the tip of the CVC should stand in the SVC have shown remarkable results; these landmarks are the edge of the right transverse process of the first thoracic spine (T1) and the center of carina (C). The distance between these two is termed as the C-length. The C-length can be identified by drawing a circle (compass method) where its focal point is the carina (C) and the edge of the circle passing over the edge of the right transverse process of the T1; the length of the radius will be the size of the C-Length (Figure 1). In this method PA CXRs preceding the operation were taken and assessed for the initial measurements of the C-length and compared with the post-operative PA CXRs for evaluation.^10^

Our aim in this study was to compare the conventional 15 cm method^15^ with the C-length method for the accurate placement of tip of the CVC in the SVC after insertion through the right internal jugular vein.

## Materials and Methods

A calculation was done for the estimation of the sample size, the right internal jugular vein catheters’ tips mean distance to the carina was chosen from a previous study (4) for the purpose of the power analysis (α = 0.05, β = 0.2; Ζ_α/2_ = 1.94 Ζ_β_ = 0.84, Ѕ1=Ѕ2= 0.9, μ_1_- μ_2 = 0.6_) a number of 100 for each group was obtained.

n=4(Za2+Zβ)2σ2(μ1−μ2)2

**Figure 2:**
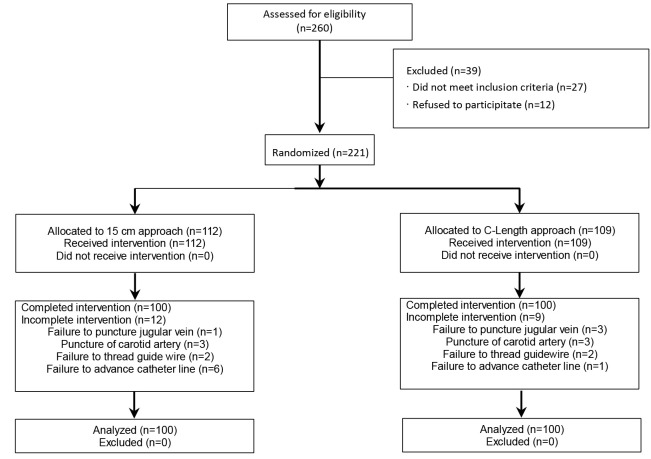
Consort flow diagram.

The subjects were recruited from the adult patients who were referred to Shahid Mohammadi Hospital, affiliated to Hormozgan University of Medical Sciences in Bandar Abbas, Iran; in year 2013 for elective coronary artery bypass graft (CABG) surgery. A written informed consent was obtained from all patients. Due to drop outs a total of 260 patients were recruited, ineligible cases were omitted and the rest were randomly divided into two groups by the use of random box of card numbers. A hundred of these patients were finally analysed in each group prospectively. The patients who had an ejection fraction less than 30%, emergency surgery and patients with deformities of neck and chest were excluded from the study (Figure 2).

The general anesthesia and monitoring techniques applied were similar for all patients. The patients were placed in 15 degrees Trendelenburg position with a slight left rotation of the head. The triangle between the sternal and clavicular head of the sternocleidomastoid muscle was used as a landmark for right internal jugular vein (RIJV) catheterization. The skin was prepared in a sterile fashion with povidone-iodine. After preparation and draping of the site of insertion, the Seldinger technique was applied for cannulation of the RIJV starting with localizing the vein with a ‘finder’ needle connected to a 2-ml syringe, directed towards the right nipple with a 45° angle to the skin. The return of venous blood into the syringe attached to the needle confirmed entry into the vessel, and the finder needle was used to guide a 19-gauge, 10-cm needle connected to a 5-ml syringe, then a 20-cm long, triple-lumen CVC (ARROW Multi-lumen CVC set with Blue Flextip^®^ Catheter, REFCV-15703, Arrow International Inc., 2400 Bernville Rood, Reading, PA 19605 USA) was introduced into the RIJV, after the guide wire insertion the patients were returned to the supine position and his/her head and neck were placed in the neutral position. The CVCs were introduced and fixed according to the predetermined lengths for each group. In the first group the depth of catheter insertion method was by fixing the catheter at the 15 centimetre point (at the marked indicator on its shaft length of CVC), at the skin insertion hole and in the second group the length of fixation was defined by using the C-Length method as described above. In C-Length group, the C-Length before the insertion of catheter was identified according to the preoperative PA CXR, thereafter the location of the tip of the catheter was assessed by a postoperative PA CXR taken later in the cardiovascular surgery intensive care unit for both groups.

The proximal SVC descends posterior to the atrial appendage to empty into the atrium through a thickened ring of tissue formed by the crista terminalis anteriorly and the crista dividens posteriorly. The two cristae constitute the embryonic transition from the sinus venosus to the true atrium and thus form the anatomic cavoatrial junction. Additionally Baskin at al have reported the mean cavoatrial junction position was 1.6 cm ±1.1 below the right heart border, 2.9 cm ±1.3 below the right main stem bronchus, and 4.0 cm± 1.0 below the carina.^16^ Accordingly a mean distance of 3 cm was considered the point of cavoatrial junction below the carina.^10^ To be justified for the location of CVC tip many other authors have also emphasized that the catheter tip must be in the upper SVC.^17-19^

Baskin et al also demonstrated that the origin of the SVC is only 3 cm superior to the carina,^16^ as a result we considered a distance of 3 cm above carina the upper SVC zone where the risk of cardiac tamponade is the least.

Based on these figures three zones were defined on the PA CXRs. The carina in the PACXR was marked by a horizontal line (the central carinal line as line C), parallel to this an arbitrary line 3 cm above the carina was considered as line U (Upper line) and 3cm under it as line L (Lower line) and the C-Length was measured as the distance between the transverse processes of T1 vertebra to the carina on the PA CXR as mentioned above. The distance between line C and line U was considered as the upper zone of SVC and the distance between line C and line L (cavoatrial junction) was considered as lower zone of SVC and below line L is the beginning of the right atrium at the end of SVC and was defined as the RA (unsafe) zone (Figure 1).The position of the CVC tip, in relation to the carina, was confirmed and measured on a postoperative full-inspiration chest radiograph (CXR) from the Picture Archiving & Communication System (PACS, iQ-WEBX, IMAGE Information Systems Co., Ltd., London, UK). CVC tips positioned above the carina level were presented in negative values, and those below the carina were presented in positive values.

Demographic information and hemodynamic parameters were documented. Chi-square and *t* tests were used for statistical analysis (SPSS software), and a *P* value <0.05 was considered significant.

## Results

A total of 260 patients who underwent CABG surgery were enrolled in to the study from which 200 remained for analysis (Figure 2). From these patients 60% (n = 120) were male and 40% (n = 80) were female. The results of the demographical data investigation and vital signs of patients are shown in Table 1.

**Table 1:** Comparison of the demographic and hemodynamic parameter values between C-Length group and 15 cm group

	**C-Length group**	**15 cm group**	***P*** ** value**
	**Mean ± SD**	**Mean ± SD**	
Age (y)	59.76±11.95	57.38±11.32	0.121
Weight (kg)	62.30±11.66	58.14±11.12	0.260
EF (%)	50.00±9.22	50.61± 12.03	0.615
Height (cm)	163.93±9.25	163.00±8.61	0.911
Central venous pressure (mm Hg)	13.09±16.09	9.97±3.49	0.199
Systolic blood pressure (mm Hg)	148.60±23.75	152.38±38.60	0.512
Diastolic blood pressure (mm Hg)	70.98±11.30	73.14±12.85	0.655
Heart rate (bpm)	87.17±16.16	79.96±17.50	0.290
SaO2 (%)	96.56±12.55	98.06±1.36	0.098

In 15 cm group the average distance of catheter tip to skin was 15±0.56 cm and all catheters (100%) were below the line C. In 22 cases (22%) the catheter tips were located in less than 3 cm below line C (Lower SVC zone) and in 78 cases (78%) the catheter tips were more than 3 cm below line C (RA zone). The average distance of catheter tip to line C in this group was (+4.22 ± 2.10 cm) (+ means below carina) (Table 2).

**Table 2:** Comparison of the catheter tip location zones between the C-length and 15 cm groups

	**Location**								***P*** ** value**
	**Above C line**			**On C line**		**Below C line**			
	**Upper SVC zone**		**Carina**		**Lower SVC zone**		**RA zone**		
	%	No.	%	No.	%	No.	%	No.		
15 cm group	0	0	0	0	22	22	78	78	<0.001
C-Length group	14	14	34	34	52	52	0	0	
Total	14	14	34	34	74	74	78	78	

In C-length group the mean distance of the catheter tip to skin was 10.77±1.72 cm and in 34 cases (34%) the catheter tips were located on line C and in 14 cases (14%) they were above line C. The average distance of catheter tips to line C was (-1.14 ± 0.69 cm) (- means above carina). These results mean that in the C-Length group 48 (~50%) of catheter tips were certainly located in SVC at a level above the carina. In 52 cases of the C-Length group the catheter tips were located below line C (less than 3 cm); in the lower SVC zone, and the average distance of catheter tip to line C was (+0.77 ±0.5 cm) (Table 2).

There was a significant difference between two groups related to the location of the catheter tips in the SVC (*P* < 0.001). In this study a total of 152 cases from both methods had their catheter tips located below line C and the average distance to the carina was +2.38±2.98 cm (Table 3).

**Table 3:** Comparing the mean length of catheter in the SVC and the tip’s mean distance from carina between the two groups

**Mean distance of catheter tip to**	**Group 15 cm**	**Group C length**
Skin	0.56‏±15	1.72‏±10.77
Line C	2.10‏±4.22‏+	0.59‏±+0.95

## Discussion

In the present study 100% of catheter tips in the 15 cm group versus 52% of them in the C-Length group were located below the line C. In other words 48% of the catheter tips were located above the carina in the C-Length group.

In the study of Lee J and Lee Y, 57.4% of catheter tips in the 15 cm group were placed in lower SVC zone and 37.8 % of them in the RA zone, however in their C-length group 87.4% of the catheter tips were placed in the upper SVC zone and 12.6% cases were placed in the lower SVC zone and none in the RA zone.^10^ Compared to their study^10^ the average catheter tip distances to the carina were higher in our study groups (‏+4.22± 2.10 vs. +2.77 cm for 15 cm group) and (+0.95±0.59 vs. −1.42 cm for C-Length group), as a result a lower proportion of catheters tips in our study were in the upper SVC zone. Additionally in our study, none of the catheters in the 15 cm group were placed above the line C. A possible explanation for this inconsistency with their study maybe because the mean of heights of the patients in our study were lesser (163.47±8.93 vs. 164.8 ± 13.1 cm).

In the study of Ezri and colleagues^21^ they compared the 15 cm method with another topographic measurement method by drawing a line from thyroid notch to the sternal manubrium, in their 15 cm group 20% of the catheter tips were located in the RA which was lesser compared to our study (78%).

In a multi-center study by McGee and colleagues^15^ using a conventional method of estimating the approximate distance of insertion either by measuring the catheter on the patient’s chest or by operator determined preset anatomic land marks. Their result was a 47% intra-cardiac (RA zone) located CVC tips, interestingly the physicians who had inserted the catheters in their centers did not attempt to reposition them to the SVC zones, They suggested that the operators may not have been sufficiently concerned with potential complications of right atrial tip placement or not be aware of Food and Drug Administration guidelines.^7^ In our study 78% of the 15 cm group were in the RA zone and all of the C-Length group CVC catheters were in the SVC zones.

The pericardial reflection can be located at a mean distance of 5±10 mm below the carina (range, 29 mm below to 25 mm above)^21^ a finding that supports the results obtained in cadaver-based studies^22,23^ in which the pericardial reflection always ended below the carina. However, it was shown that 30% of the patients had a pericardium ending above the carina, with a maximum distance of 25 mm. It was concluded that the best results in terms of CVC tip positions in the extra-pericardial SVC (the portion of SVC between the pericardial reflection and the confluence of the SVC or cephalad origin of the innominate veins) would have been achieved by using 85% of the sternoclavicular joint-to-carina distance (in 86% of their patients) or by positioning the CVC tip 9 mm above the carina (in 84% of their patients).^21^ Our study had been conducted before the publication of Dulce and colleagues work^21^ and their results need further confirmation. A possibility of 30% of patients having a pericardial reflection above carina is new interesting information and may cause concern over the hypothesis of our study which is in accordance to many other previously reported results and opinions of other researches.^3,4,24,25^ Carina can be considered as a radiologic landmark on a CXR and the CVC tip should be above it otherwise the tip could be in the pericardial sac,^23^ additionally this location can impose a risk for thrombosis or, perforation especially for left sided CVCs because the SVC is only 6 cm long and the carina is about 3.5 cm above the SVC/atrial junction. Besides the PA CXR does not identify small vessel location like azygus vein or an extra vascularly placed CVC near to the correct position.^17^ For this reason applying the 15 cm traditional method for all patients either right or left sided catheter placement can be hazardous. We had all the CVCs inserted from the right internal jugular vein and we did not have any serious complications.

Additionally it has been postulated that the CVC retains its curvature as contained in the manufactured box and because of that predicting the depth of catheter placement with the CVC itself was a recommended approach^5^ however in our study the C-Length was measured as a straight line. Considering the adaptive curvature together with the C-Length could provide an alternative approach for a safe CVC placement. According to our findings and the above studies more investigations are needed for a safer management of the central venous cannulation procedures applied to the pericardial vascular area.

## Conclusion

Considering that the majority of placements in C-Length method were located in SVC and almost all of the placements in 15 cm method were located in lower SVC zone or RA zone, we conclude that the C-Length method is more reliable in estimating the correct location of the catheter tip in comparison with the conventional 15 cm method. Therefore to prevent the inappropriate placement of catheter and its possible complications the C–Length method is safer and the preferable approach than the conventional 15 cm method. Ultimately the CVC placement with a 15 cm traditional method is not suitable for all cases due to differences in height and body structures so that a more individualized approach is needed to attenuate its shortcomings.

## Ethical approval

Ethical Principles for Medical Research Involving Human Subjects outlined in the Declaration of Helsinki were applied to the study design. This study was approved by Anesthesiology, Intensive Care and Pain Management Research Center, and Deputy for Research and Medical Ethics Committee of Hormozgan University of Medical Sciences (No. HEC-92-4-25-1). The study protocol was registered in the Iranian Registry of Clinical Trials website (identifier: IRCT2015071418091N4; https://www.irct.ir/trial/16503).

## Competing interests

We declare that none of the authors have any financial interests or assets to any commercial firm, organization, company or individual that could create a situation of conflict with the content and results of this study or manuscript.

## Funding/Support

This study was supported by Hormozgan University of Medical Sciences, the Deputy of Research of Hormozgan University of Medical Sciences and Anesthesiology, Critical Care and Pain Management Research Center of Hormozgan University of Medical Sciences.

## Acknowledgment

We would like to thank the Hormozgan Anaesthesiology, Critical Care and Pain Management Research Center staff Mrs Farnoosh Towfighi and Mrs. Nina Heydari, also Dr. Maziyar Jamshidi and Mr. Mehrab YarAlahi from the Aftab Institute for the graphic works. We would like to also acknowledge the Shahid Mohamadi Hospital Digital Radiology staff Mr. Hassan Rahnama and Mrs. Maryam Javadi for helping for the chest x rays, and our statistics adviser Mrs. Shideh Rafati. We are grateful to the Deputy of Research Center of Hormozgan University of Medical Sciences that had provided the financial and official support to the study. We are also sincerely thankful to our counsellors in Clinical Research Development Center of Shahid Mohammadi Hospital.
